# Retraction: Relationship between Resting Pulse Rate and Lipid Metabolic Dysfunctions in Chinese Adults Living in Rural Areas

**DOI:** 10.1371/journal.pone.0249845

**Published:** 2021-04-06

**Authors:** The PLOS ONE Editors

Following the publication of this article [1], the corresponding author requested its retraction. The corresponding author stated that the article’s authors do not own the data used in the study and did not obtain permission from the data owners to publish the reported results.

In light of the data ownership issues, the PLOS ONE Editors retract the article.

CJW, YQL, LLL, LW, AGY, YRG, and WJL agreed with the retraction. JZZ either did not respond directly or could not be reached.
